# Impact of Contextual Factors on External Load During a Congested-Fixture Tournament in Elite U’18 Basketball Players

**DOI:** 10.3389/fpsyg.2019.01100

**Published:** 2019-05-15

**Authors:** José Pino-Ortega, Daniel Rojas-Valverde, Carlos David Gómez-Carmona, Alejandro Bastida-Castillo, Alejandro Hernández-Belmonte, Javier García-Rubio, Fábio Yuzo Nakamura, Sergio José Ibáñez

**Affiliations:** ^1^Departamento de Actividad Física y Deporte, Facultad de Ciencias del Deporte, Universidad de Murcia, San Javier, Spain; ^2^Centro de Investigación y Diagnóstico en Salud y Deporte (CIDISAD), Escuela de Ciencias del Movimiento Humano y Calidad de Vida, Universidad Nacional, Heredia, Costa Rica; ^3^Grupo de Optimización del Entrenamiento y el Rendimiento Deportivo (GOERD), Facultad de Ciencias del Deporte, Universidad de Extremadura, Cáceres, Spain; ^4^Department of Medicine and Aging Sciences, Università degli Studi “G. d’Annunzio” Chieti – Pescara, Chieti, Italy; ^5^College of Healthcare Sciences, James Cook University, Townsville, QLD, Australia; ^6^Associate Graduate Program in Physical Education, Federal University of Paraíba, João Pessoa, Brazil

**Keywords:** team sports, external load, kinematics, indoor, ultra-wide band

## Abstract

An understanding of basketball physical demands during official matches is fundamental for designing specific training, tactical, and strategic plans as well as recovery methods during congested fixture periods. Such assessments can be performed using wearable indoor time motion tracking systems. The purpose of this study was to analyze the time-motion profile of under 18-years of age (U’18) basketball players and compare their physical demands in relation to team ranking, playing position, match periods and consecutive matches during a 7-day tournament. Relative Distance (RD), percentage of High-Intensity Running (%HIR), Player Load (PL), Acceleration (Acc), Deceleration (Dec), Peak Speed (PSpeed), and Peak Acceleration (PAcc) were recorded from 94 players (13 centers, 47 forwards, and 34 guards) belonging to eight elite teams (age: 17.6 ± 0.8 years; height: 1.91 ± 0.08 m; body mass: 82.5 ± 8.8 kg). WIMU PRO^TM^ inertial measurement units with ultra-wide band (UWB) indoor-tracking technology recorded 13 matches during the *Adidas Next Generation Tournament Finals* in the 2016–2017 season. Paired *t*-tests and one-way analyses of variance with omega partial squared ($\omega_{p}^{2}$) and Cohen’s effect sizes (*d*) were used to analyze for differences between variables. According to team quality, the best teams had lower RD (*p* = 0.04; *d* = −0.14). Guards presented higher RD (*p* < 0.01; $\omega_{p}^{2}$ = 0.03), PSpeed (*p* < 0.01; $\omega_{p}^{2}$ = 0.01) and PAcc (*p* < 0.01; $\omega_{p}^{2}$ = 0.02) compared to forwards and centers. The first quarter showed differences with higher RD (*p* < 0.01; $\omega_{p}^{2}$ = 0.03), %HIR (*p* < 0.01; $\omega_{p}^{2}$ = 0.02), and PL (*p* < 0.01; $\omega_{p}^{2}$ = 0.04) compared to all other quarters. The third match of the tournament presented higher demands in RD (*p* < 0.01; $\omega_{p}^{2}$ = 0.03), HIR (*p* < 0.01; $\omega_{p}^{2}$ = 0.01) and PL (*p* < 0.01; $\omega_{p}^{2}$ = 0.02) compared with the first two matches. This study showed that team quality, playing position, match period, and consecutive matches throughout an U’18 basketball tournament influenced the kinematic demands experienced by players during official competition. Therefore, each of these contextual factors should be considered in managing the load and developing individualized strategies for players in tournament settings.

## Introduction

Basketball is considered as a team sport involving intermittent efforts due to the elevated number of instances of high-intensity running alternating with low-intensity periods (Stojanović et al., 2018). Currently, most elite team sport players are exposed to congested fixtures with a high number of matches or competitions within a few days (Ibáñez et al., 2009; Dellal et al., 2013; Rojas-Valverde et al., 2018), and this kind of situation could lead to an increase in fatigue and injury risk (McLean et al., 2018). In fact, in recent years this competitive dynamic has increased the interest of teams’ medical and technical staffs to analyze and thus better understand the internal and external physical load of players using objective methods during training and competition (Fox et al., 2017).

Internal load is the physiological reaction and stress experienced when faced with a stimulus (Fox et al., 2018), and it can be measured by heart rate telemetry, rating of perceived exertion, fitness-wellness tests, as well as metabolically, using biochemical, hormonal, and immunological markers (Akubat et al., 2014). On the other hand, external load is considered as the total locomotor and mechanical stress produced by an activity. Load parameters vary among brands or device version (Aughey, 2011; Cummins et al., 2013; Dellaserra et al., 2014), most of them measure: (i) distance covered per minute (m/min), (ii) average speed as an indicator of intensity of movement (km/h); (iii) percentage of high-intensity actions (% HIA), (iv) accelerations and decelerations per minute (acc/min; dec/min), and (v) impacts at different intensities or specific formulas such as PlayerLoad^TM^ (PL^TM^) (Edwards et al., 2018; Staunton et al., 2018; Svilar et al., 2018).

Analytic techniques have been used previously, employing subjective means, to classify the form and intensity of the activities in order to assess load demands in basketball (Abdelkrim et al., 2007; Matthew and Delextrat, 2009). These procedures could not be so precise and depended on the quality of video capture, the relative size and occlusion frequency of people, and also changes in illumination (Barris and Button, 2008). Standardization in the use of external load measurements, as well as the technological development of tracking systems, have allowed time-motion variables to become one of the most common methods to assess the demands of sport tasks, training sessions and official matches (Fernandez et al., 2016). New tracking technologies using local positioning systems allow the assessment of physical (Ogris et al., 2012; Leser et al., 2014; Bastida Castillo et al., 2018), accelerometrical (Boyd et al., 2011; Gómez-Carmona et al., 2018) and tactical demands (Bastida-Castillo et al., 2019a) in team sports such as basketball in indoor conditions.

Given these possibilities, current hot topics in research are focused on a better understanding of the physical and physiological demands during training and competition in basketball and the effect of contextual variables, including: (i) type of session, higher demands have been reported in official matches compared to training or simulated competition (Fox et al., 2018; Reina et al., 2018); (ii) playing position, guards usually sustained greater workloads than forwards and centers (Abdelkrim et al., 2007; Puente et al., 2017); (iii) match periods, there is evidence of a decrease in physical performance throughout the match quarters (Scanlan et al., 2015b; Staunton et al., 2018); (iv) gender, women develop higher volume loads and men higher intensity demands (Scanlan et al., 2015a); (v) players’ levels, the higher-level players performed greater intensity movements while the lower-level players covered a greater volume of distance (Scanlan et al., 2011); (vi) congested fixture periods, there is a higher demand in a competitive period with two matches per week with respect to 1 match per week (Conte et al., 2018). These contextual variables make it possible to establish the specific profile of basketball demands for a better understanding and individualization of training load (Scanlan et al., 2015b).

For these reasons, due to the current characteristics of basketball tournaments with consecutive matches (Ibáñez et al., 2009), considering the key role of intensity as a determinant of performance in team sports (Hills et al., 2018), and also the specific demands of young players at the physical (Oba and Okuda, 2008), technical-tactical (García et al., 2010) level, and relative age effect (Arrieta et al., 2016) compared to adult players, the aims of this study were to: (1) describe the intensity time-motion profile of elite U’18 basketball players and (2) compare their demands in relation to team quality, playing position, match periods and three consecutive matches during an international tournament characterized by congested fixtures.

## Materials and Methods

### Design

A cross sectional design with natural groups was employed in the current study (Ato et al., 2013) to analyze the intensity time-motion profile of elite U’18 basketball players during the *Adidas Next Generation Tournament* (ANGT 16-17) using an ultra-wide band (UWB) tracking system.

**Table 1:** Anthropometric characteristics of the participants by playing position.

Variable	All participants (*n* = 94)	Guard (*n* = 34)	Forward (*n* = 47)	Center (*n* = 13)
Age (years)	17.6 ± 0.8	17.6 ± 0.8	17.6 ± 0.3	17.5 ± 0.5
Height (m)	1.91 ± 0.08	1.93 ± 0.03	1.89 ± 0.05	1.86 ± 0.04
Body mass (kg)	82.5 ± 8.8	88.2 ± 9.8	78.4 ± 7.1	76.2 ± 5.4
BMI (kg/m^2^)	22.7 ± 1.8	24.4 ± 1.8	23.4 ± 1.2	22.5 ± 1

*BMI, body mass index; data expressed as mean ± SD.*

### Participants

A total of 94 elite under 18-year-old basketball players (see Table 1), members of eight teams, were studied during the 13^th^ edition of the Euroleague Basketball *ANGT* finals held from May 18 to 21, 2017.

The teams’ staffs and tournament managers gave their consent for participation in this research^1^. As all players were over 16 years old, they signed a written consent before the tournament started to give their assent for participation without needing their parents’ permission, and approval was given by the Bioethics Commission of the University (Reg. Code 67/2017). The study was conducted according to the Declaration of Helsinki (World Medical Association, 2013) guidelines.

### Instruments

To collect time-motion pattern data measurements, all players were equipped with an inertial measurement unit (IMU) with UWB tracking system technology (WIMUPRO^TM^, RealTrack Systems, Almería, Spain). The sampling frequency for positioning and for accelerometer load was 18 and 100 Hz, respectively. The accuracy (x-axis = 5.2 ± 3.1 cm; y-axis 5.8 ± 2.3 cm) and reliability (x-axis, ICC = 0.65; y-axis, ICC = 0.85) of the indoor tracking system technology on the tournament court has been previous reported in different courses (perimeter, middle line, paint lines, center circle, and 6.75-m line) at a speed of over 15 km/h (Bastida-Castillo et al., 2019b). In addition, the within and between-units reliability of accelerometers in: (a) laboratory (static: with and without stress; dynamic: 10 and 30 Hz vibrations; coefficient of variation = 0.23–0.78%) and (b) field conditions (incremental running treadmill test, coefficient of variation = 2.20%; and SAFT^90^, coefficient of variation = 2.96%), with (c) the test–retest reliability (*p* = 0.46–0.98; *t* = 0.01–0.73; *r* = 0.86–0.96) has also been analyzed (Gómez-Carmona et al., 2018).

### Variables

#### Time Motion Analysis

In order to compare results among playing positions, quarters, matches and team quality; variables were selected related to playing time per minute: (a) Relative Distance (RD, m/min); High Intensity Running (HIR, % of total distance covered at over 16 km/h); (c) Player Load, accumulated accelerometer load in the three axes of movement (PL, a.u./min); Total Accelerations (Acc, count/min) and Decelerations (Dec, count/min) and Peak Speed (PSpeed, km/h) and Peak Acceleration (PAcc, m/s^2^) (Vazquez-Guerrero et al., 2018).

#### Team Quality

The teams which took part of the tournament (in order of final positions) were: CFBB Paris (CFBBP), KK Mega-Bemax-Belgrade (MBB), PBC CSKA Moscow (CSKAM), Real Madrid (RM), FC Barcelona-Lassa (FCBL), Fenerbache Istanbul (FI), KK Crvena Zvezda (CZ), and Žalgiris Kaunas (ZK). For further analysis, the teams were divided by the final tournament ranking into two groups as follow: best teams (1^st^-to-4^th^) (*n* = 513) vs. worst teams (5^th^-to-8^th^) (*n* = 521).

#### Playing Position

In order to explore differences by playing positions the total sample was grouped in the three regular basketball roles: 13 centers (*n* = 154), 47 forwards (*n* = 466), and 34 guards (*n* = 374).

#### Match Period

Data from each match were divided into four periods according to official basketball rules: quarter 1 (Q1; *n* = 263), quarter 2 (Q2; *n* = 269), quarter 3 (Q3; *n* = 249), and quarter 4 (Q4; *n* = 253).

#### Consecutive Matches Throughout the Tournament

**FIGURE 1:**
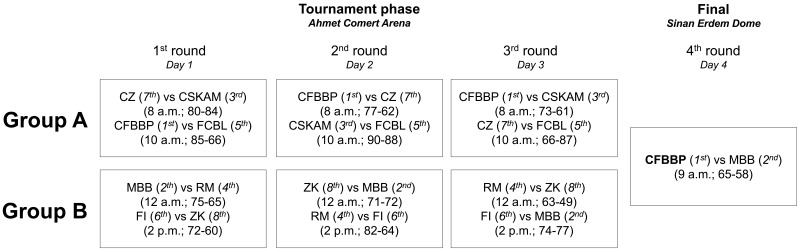
Diagram to show the match distribution throughout the tournament.

The final round of the ANGT 16-17 was composed of 13 matches that were divided into four rounds. The first, second and third round were part of the Tournament phase, and the fourth round was the final match of the championship. Each round of the tournament phase was composed of four matches (two matches in group A and two matches in group B) (Figure 1). The sample analyzed in each round was: round 1 (*n* = 292), round 2 (*n* = 327), and round 3 (*n* = 415); and the last one in the final round (*n* = 58). The final round has not been considered for analysis as only two teams participated.

### Procedures

The tournament lasted 4 days. The eight teams were randomly divided into two groups, all teams played against each other in each group and the first placed team in group A and B at the end of the round played the final. There was a total of 13 matches, and 915 records were obtained from the players. Matches in the tournament round were played in the “Ahmet Comert Arena,” except the final round which was played in the “Sinan Erdem Dome” stadium (Istanbul, Turkey) (see Figure 1 for more details). The matches in the tournament round were randomly held between 8 am and 2 pm; and the final round at 9 am.

**FIGURE 2:**
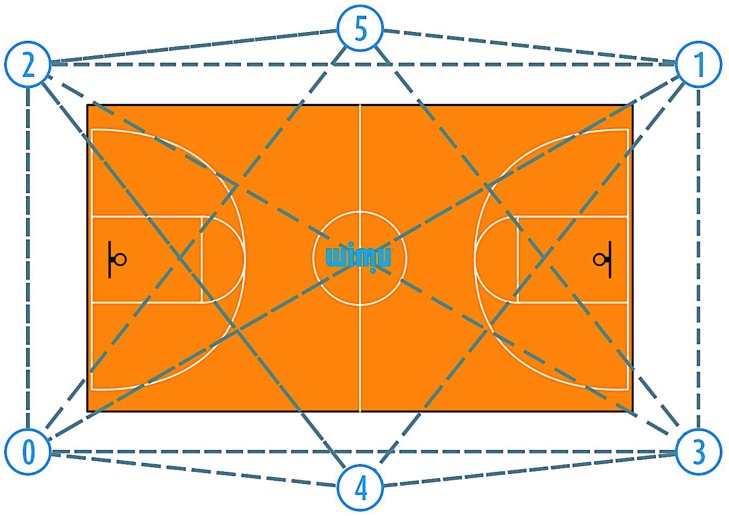
Ultra-Wide Band Antennae court setting/positions.

The IMU devices were calibrated and the UWB system was installed around the court following a previous study protocol (Bastida Castillo et al., 2018). Firstly, the UWB system was installed on the field as follows: (i) six antennae with UWB technology were fixed 4.5 m from the perimeter line of the field, except for the ones located in the middle line of the field that were fixed at 5.5 m, in this way the antennae formed a hexagon for a better emission and reception of the signal (see Figure 2). All of them were located at a height of 3 m and held by a tripod; (ii) once installed, they were switched on one by one making sure that the master antenna was the last, and then a process of autocalibration of the antennae was carried out for 5′; (iii) in a last step, the tracking devices were switched on and a process of recognition and automatic communication with the antennae was carried out during 1′.

Before the match started, IMUs were placed into a specific custom neoprene vest located on the middle line between the scapulae at the C7 level, fitted tightly to the body as is typically used in matches (Svilar et al., 2018; Vazquez-Guerrero et al., 2018).

Raw time-motion data were downloaded and exported in excel format using S PRO specialized software. The players’ roster was obtained from the official championship webpage^2^ and cross checked with the team staff. The criterion to include players in the statistical analysis was participation in >60% of total playing time per quarter, except time-outs and between-quarter breaks. All within quarter breaks were considered in the analysis (e.g., free-throw, fouls, ball out, changes, and others) in order to explore natural match behavior. This criterion was employed to homogenize the sample considering the player’s match participation, especially when the analyzed variables represented the intensity of playing actions (Sampaio et al., 2010).

### Statistical Analysis

Mean and standard deviation (*M* ±*SD*) of the variables were used to describe the data in the four different variables. Data normal distribution was confirmed using the Kolgomorov–Smirnov test and the homogeneity of variance assumption was made using the Levene Test. Firstly, an independent *t*-test was performed to compare best (1^st^-to-4^th^) and worst teams (5^th^-to-8^th^) considering their final tournament positions. Paired magnitude of differences was qualitatively interpreted using Cohen’s effect sizes (*d*) as follows: *d* > 0.2 as *small*, *d* > 0.5 as *moderate*, and *d* > 0.8 as *large* effect size (Cohen, 1988). Three different one-way analyses of variance were performed in order to compare means of PL, HIR, RD, Acc, Dec, PSpeed, and PAcc by (1) playing position, (2) match periods, and (3) consecutive matches throughout the tournament. The magnitude of the differences was qualitatively interpreted using partial omega squared ($\omega_{p}^{2}$) as follows: >0.01 *small*; >0.06 *moderate*, and >0.14 *large* (Cohen, 1988). Alpha was prior set at *p* < 0.05. The data analysis was performed using Statistical Package for the Social Sciences (SPSS Statistics, release 22, IBM Corporation, Chicago, IL, United States) and plot design using GraphPad Prism (release 7, La Jolla, CA, United States).

**Table 2:** Descriptive data (means ± SDs; 95% CIs, in parentheses) of teams by final tournament ranking.

Team ranking	Relative distance (m/min)	High intensity running (%)	Player load (a.u./min)	Acceleration (count/min)	Deceleration (count/min)	Peak speed (km/h)	Peak acceleration (m/s^2^)
1^st^	73.9 ± 15.2	4.1 ± 2.9	1.5 ± 0.4	16.1 ± 2.5	15.6 ± 2.6	18.7 ± 3.6	3.4 ± 0.6
	(71.2–76.8)	(3.6–4.6)	(1.4–1.6)	(15.6–16.5)	(15.1–16.1)	(18–19.4)	(3.3–3.5)
2^nd^	71.6 ± 10.7	3.5 ± 2.2	1.3 ± 0.2	16.4 ± 1.3	16 ± 1.3	19.2 ± 2.2	3.2 ± 0.4
	(69.5–73.7)	(3.1–3.9)	(1.3–1.4)	(16.2–16.7)	(15.8–16.2)	(18.8–19.6)	(3.2–3.3)
3^rd^	67.4 ± 16.7	3.5 ± 2.6	1.3 ± 0.3	16 ± 3.5	15.5 ± 3.6	18 ± 4.9	3.2 ± 0.6
	(64.4–∖70.4)	(3.1–4)	(1.2–1.3)	(15.4–16.6)	(14.9–16.2)	(17.1–18.8)	(3.1–3.3)
4^th^	71.7 ± 11.5	3.2 ± 2.3	1.4 ± 0.2	16.5 ± 1.8	16.1 ± 1.8	18.6 ± 3.4	3.1 ± 0.4
	(69.5–74)	(2.7–3.6)	(1.3–1.4)	(16.1–16.8)	(15.8–16.5)	(17.9–19.3)	(3.1–3.2)
5^th^	79.2 ± 13.2	3.8 ± 2.2	1.4 ± 0.2	16.9 ± 1.3	16.5 ± 1.3	18.8 ± 2.8	3.4 ± 0.3
	(76.7–81.7)	(3.4–4.2)	(1.3–1.4)	(16.6–17.1)	(16.2–16.7)	(18.3–19.4)	(3.3–3.4)
6^th^	74.4 ± 17.4	3.9 ± 2.9	1.4 ± 0.3	16 ± 2.6	15.5 ± 2.5	18.5 ± 3.5	3.3 ± 0.6
	(71.3–77.5)	(3.4–4.4)	(1.4–1.5)	(15.6–16.5)	(15.1–16)	(17.9–19.1)	(3.2–3.4)
7^th^	70.6 ± 16.3	3.3 ± 2.5	1.3 ± 0.3	16.2 ± 2.1	15.8 ± 2.2	18.3 ± 3.6	3.3 ± 0.6
	(67.6–73.7)	(2.8–3.7)	(1.2–1.3)	(15.8–16.6)	(15.3–16.2)	(17.6–19)	(3.1–3.4)
8^th^	68.9 ± 17.2	3.1 ± 2.2	1.2 ± 0.3	15.9 ± 2.8	15.5 ± 2.7	17.6 ± 4.8	3.1 ± 0.6
	(65.8–72.1)	(2.7–3.5)	(1.2–1.3)	(15.4–16.4)	(15–16)	(17.7–18.5)	(3–3.3)

## Results

### Team Quality

**FIGURE 3:**
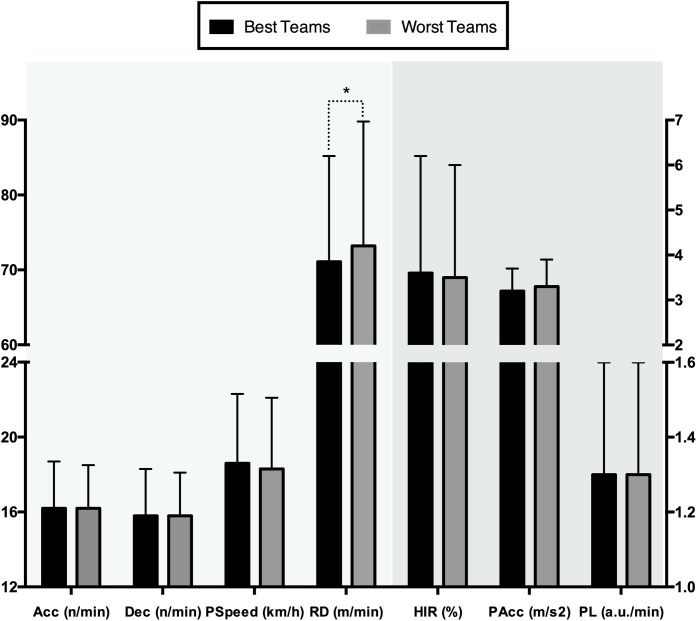
Intensity variables by final tournament ranking (best four teams vs. worst four teams). ^∗^*p* < 0.05.

Table 2 shows the descriptive data of the variables analyzed per team in order of their final standings. When comparing best teams (1^st^-to-4^th^) vs. worst teams (5^th^-to-8^th^), differences with a small effect size were found in: RD (*t* = −2.09, *p* = 0.04; *d* = −0.14 *small effect*). There were no statistical differences in HIR (*t* = 0.42, *p* = 0.67, *d* = 0.03), PL (*t* = 0.48, *p* = 0.63, *d* = 0.03), Acc (*t* = −0.12, *p* = 0.90, *d* = 0.01), Dec (*t* = 0.01, *p* = 0.99, *d* = 0), PSpeed (*t* = 1.17, *p* = 0.24, *d* = 0.08) and PAcc (*t* = −0.74, *p* = 0.46, *d* = −0.05). the best teams had a significant lower RD (see Figure 3).

**Table 3:** Means ± SDs, 95% *Cis* (in parentheses), one-way ANOVA and pair-wise comparisons with Cohen’s effect sizes (d) of basketball time-motion studied demands per playing positions.

Playing position	Guards	Forwards	Centers
**Relative distance (m/min)**	73.9 ± 13.9^¶¶^	72.2 ± 15.3^¶^	66.14 ± 19.8^∗∗†^
	(72.4–75.5)	(70.7–73.6)	(61.5–69.8)
High intensity running (%)	3.5 ± 2.4	3.6 ± 2.7	3.3 ± 2.9
	(3.3–3.8)	(3.4–3.9)	(2.8–3.9)
Player load (a.u./min)	1.4 ± 0.3	1.3 ± 0.3	1.3 ± 0.4
	(1.3–1.4)	(1.3–1.4)	(1.2–1.4)
Acceleration (count/min)	16.3 ± 2.2	16.1 ± 1.9	16.3 ± 4.2
	(16.1–16.6)	(16–16.3)	(15.5–17.1)
Deceleration (count/min)	15.9 ± 2.2	15.7 ± 2	15.9 ± 4.2
	(15.6–16.1)	(15.5–15.9)	(15.1–16.6)
**Peak speed (km/h)**	18.8 ± 3.3^¶^	18.4 ± 3.7^¶^	17.2 ± 5.2^∗†^
	(18.5–19.2)	(18.1–18.8)	(16.3–18.2)
**Peak acceleration (m/s^2^)**	3.4 ± 0.5^¶¶¶†^	3.3 ± 0.5^∗¶¶^	2.9 ± 0.7^∗∗∗††^
	(3.3–3.4)	(3.3–3.4)	(2.8–3.1)

*ANOVA statistical differences (bold text) (p < 0.05). ^∗^Statistical differences with guards (p < 0.05; effect size: ^∗^small, ^∗∗^moderate, ^∗∗∗^large). ^†^Statistical differences with forwards (p < 0.05; effect size: ^†^small, ^††^moderate). ^¶^Statistical differences with centers (p < 0.05; effect size: ^¶^small, ^¶¶^moderate, ^¶¶¶^large).*

### Playing Position

Differences between playing positions were evident in RD (*F* = 10.76, *p* < 0.01, $\omega_{p}^{2}$ = 0.03, *small effect*), PSpeed (*F* = 7.59, *p* < 0.01, $\omega_{p}^{2}$ = 0.02, *small effect*) and PAcc (*F* = 27.23, *p* < 0.01, $\omega_{p}^{2}$ = 0.06, *small effect*); but there were no differences in HIR (*F* = 0.44, *p* = 0.65, $\omega_{p}^{2}$ = 0), PL (*F* = 2.36, *p* = 0.09, $\omega_{p}^{2}$ = 0), Acc (*F* = 0.52, *p* = 0.60, $\omega_{p}^{2}$ = 0) and Dec (*F* = 0.39, *p* = 0.68, $\omega_{p}^{2}$ = 0). Guards presented higher RD, PSpeed and PAcc (*p* < 0.01; guards > forwards > centers). In addition, forwards presented higher RD, PSpeed and PAcc than guards (*p* < 0.01) (see Table 3).

### Match Periods

There were differences among match periods with a small to moderate effect size in RD (*F* = 9.82, *p* < 0.01, $\omega_{p}^{2}$ = 0.03 *small effect*), HIR (*F* = 7.19, *p* < 0.01, $\omega_{p}^{2}$ = 0.02 *small effect*) and PL (*F* = 12.87, *p* < 0.01, $\omega_{p}^{2}$ = 0.04 *small effect*). No differences were found in Acc (*F* = 1.76, *p* = 0.15, $\omega_{p}^{2}$ = 0.01), Dec (*F* = 1.59, *p* = 0.19, $\omega_{p}^{2}$ = 0.01), PSpeed (*F* = 0.49, *p* = 0.69, $\omega_{p}^{2}$ = 0) or PAcc (*F* = 1.318, *p* = 0.267, $\omega_{p}^{2}$ = 0).

**Table 4:** Means ± SDs, 95% *Cis* (in parentheses), one-way ANOVA and pair-wise comparisons with Cohen’s effect sizes (d) of basketball kinematic studied variables per period.

Period	1^st^ Quarter	2^nd^ Quarter	3^rd^ Quarter	4^th^ Quarter
**Relative distance (m/min)**	75.5 ± 17.5^xx^	72.1 ± 13.4^x^	73.2 ± 13^x^	67.8 ± 16.6^∗∗†¶^
	(73.2–77.8)	(70.4–73.8)	(71.5–75)	(65.6–70)
**High intensity running (%)**	4.1 ± 2.7^x¶^	3.6 ± 2.4	3.3 ± 2.4^∗^	3.1 ± 2.4^∗^
	(3.8–4.5)	(3.3–3.9)	(3–3.6)	(2.8–3.4)
**Player load (a.u./min)**	1.4 ± 0.3^xx†^	1.3 ± 0.3^∗x^	1.4 ± 0.3^xx^	1.2 ± 0.3^∗∗†¶¶^
	(1.4–1.5)	(1.3–1.4)	(1.3–1.4)	(1.2–1.3)
Acceleration (count/min)	16.1 ± 2.6	16.4 ± 2.4	16.4 ± 1.7	16 ± 2.8
	(15.8–16.5)	(16.1–16.7)	(16.2–16.6)	(15.6–16.4)
Deceleration (count/min)	15.7 ± 2.6	15.9 ± 2.4	16 ± 1.7	15.6 ± 2.8
	(15.3–16)	(15.6–16.2)	(15.8–16.2)	(15.2–16)
Peak speed (km/h)	18.4 ± 4.2	18.6 ± 3.2	18.5 ± 3.5	18.2 ± 4.1
	(17.8–18.9)	(18.2–19)	(18–19)	(17.7–18.8)
Peak acceleration (m/s^2^)	3.2 ± 0.6	3.3 ± 0.5	3.3 ± 0.4	3.2 ± 0.6
	(3.2–3.3)	(3.2–3.3)	(3.2–3.3)	(3.1–3.3)

*ANOVA statistical differences (bold text) (p < 0.05). ^∗^Statistical differences with 1^st^ quarter (p < 0.05; effect size: ^∗^small, ^∗∗^moderate). ^†^Statistical differences with 2^nd^ quarter (p < 0.05; effect size: ^†^small). ^¶^Statistical differences with 3^rd^ quarter (p < 0.05; effect size: ^¶^small, ^¶¶^moderate). ^x^Statistical differences with 4^th^ quarter (p < 0.05; effect size: ^x^small, ^xx^moderate).*

All intensity variables tended to decrease across the match periods (see Table 4). The change percentage of the first period compared to the fourth period was 10.2% in RD, 24.4% in HIR and 14.28% in PL. In a specific team analysis, the highest percentage changes between the first and fourth periods were found in the tournament champion team (RD: −17.1%; HIR: −38.9%; PL: −23.53%). The first quarter presented higher values in RD (*p* < 0.01; 1^st^ > 3^rd^ > 2^nd^ > 4^th^), HIR (*p* < 0.01; 1^st^ > 2^nd^ > 3^rd^ > 4^th^), and PL (*p* < 0.01; 1^st^ > 3^rd^ > 2^nd^ > 4^th^) compared to the rest of periods (see Table 4).

### Consecutive Matches Throughout the Tournament

**FIGURE 4:**
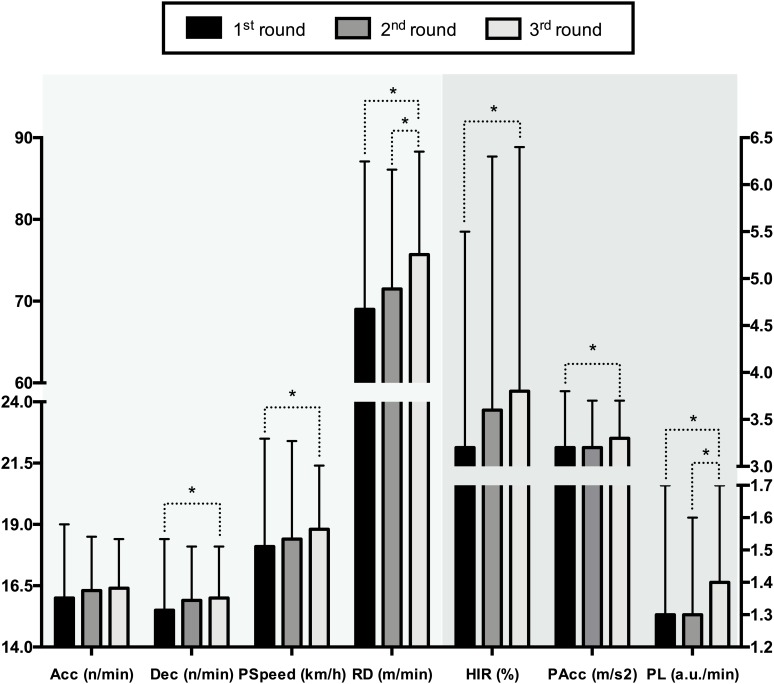
Consecutive match demands variation throughout the tournament. ^∗^*p* < 0.05.

Teams tended to increase the intensity of the match throughout the tournament. There were differences with small effect sizes between matches in RD (*F* = 14.98, *p* < 0.01, $\omega_{p}^{2}$ = 0.03), HIR (*F* = 4.95, *p* < 0.01, $\omega_{p}^{2}$ = 0.01), PL (*F* = 6.54, *p* < 0.01, $\omega_{p}^{2}$ = 0.02), Dec (*F* = 6.54, *p* = 0.04, $\omega_{p}^{2}$ = 0.02), PSpeed (*F* = 3.2, *p* = 0.04, $\omega_{p}^{2}$ = 0.01) and PAcc (*F* = 3.16, *p* = 0.04, $\omega_{p}^{2}$ = 0.01), but there was no difference in Acc (*F* = 2.45, *p* = 0.09, $\omega_{p}^{2}$ = 0), The third match presented higher RD (*p* < 0.05; 3^rd^ > 2^nd^ > 1^st^), PL (*p* < 0.05; 3^rd^ > 1^st^ > 2^nd^), Dec (*p* < 0.05; 3^rd^ > 2^nd^ > 1^st^), PSpeed (*p* < 0.05; 3^rd^ > 2^nd^ > 1^st^), and PAcc (*p* < 0.05; 3^rd^ > 2^nd^ > 1^st^) (Figure 4).

## Discussion

The objectives of this research were to identify the intensity time-motion profile of U’18 basketball players and to compare demands in relation to team quality, specific playing positions, match periods, and consecutive matches throughout the Euroleague Basketball *ANGT* 16-17 finals. The results suggested that the best teams reached a higher intensity during the matches. A decrease in demands was found over the quarters. Differences were evident in relation to playing positions, guards, and forwards performed more movements while centers received more impacts. There was an increase in the volume and high intensity demands throughout the tournament.

High-intensity running is one of the most important performance factors in team sports, and specifically in basketball (Stojanović et al., 2018). Congested fixture conditions are commonly observed during team sport tournaments (Ibáñez et al., 2009; Dellal et al., 2013; Rojas-Valverde et al., 2018), and it is important to understand their effects on physical load accumulation and performance decrement (Edwards et al., 2018).

### Team Quality

Significant between-team differences were found in RD (*p* = 0.04, *d* = −0.14 *small effect*). Despite there being no differences in variables such as PL, HIR, Acc, Dec, PSpeed and PAcc, the best teams played at a higher intensity (greater values in HIR and PSpeed), while in the rest of variables the demands between the two groups were similar. In addition, the winner of the tournament recorded the highest values in HIR (*d* = 0.07–0.39 *small effect*) and PL (*d* = 0.28–0.85 *small-to-large effect*). No previous research has studied physical demands in young players during competition from this approach. Although, in the sport science area, high-intensity activity has been analyzed in basketball during competition at different levels. In contrast to the present study, results considering the differences found in RD (best = 71.1 m/min vs. worst = 73.2 m/min), Scanlan et al. (2011) have reported differences in high-intensity running between elite (2.26% HIR) and sub-elite levels (1.93% HIR), but found no differences in total distance (6390 and 6369 m, respectively).

Similarly, Abdelkrim et al. (2010) found differences between international and national levels in moderate shuffle (14.2 and 19.8% respectively), high shuffle (9.3 vs. 8.1% respectively) and static actions (4.1 vs. 1.5% respectively). From the results obtained, the highest-level teams covered lower RD (more static actions) but performed higher intensity running, this could be explained by their greater efficiency and physical-technical-tactical level (Oba and Okuda, 2008). In this respect, previous studies have found that the best teams’ performance could be influenced by individual abilities such as experience (Kioumourtzoglou et al., 1998), technical-tactical (Ibáñez et al., 2009) and fitness player’s level (Sampaio et al., 2015).

### Playing Position Analysis

Forwards and guards recorded greater RD, PSpeed, and PAcc than centers. However, no differences among playing positions were found in HIR, PL, Acc, or Dec. This topic has been extensively studied in team sports, and specifically in basketball. For example, Scanlan et al. (2011) did not identify differences in RD by playing position (frontcourt vs. backcourt), while the present results found significance differences between forwards and guards compared to centers. On the other hand, Abdelkrim et al. (2007) reported that guards performed more high-intensity activities than forwards and centers, while the present results did not find any differences. Hence, guards recorded the higher values in HIR, in agreement with other studies (Scanlan et al., 2011, 2012). According to previous evidence (Stojanović et al., 2018) playing position particularities should be considered by basketball practitioners when planning individualized training programs, specifically in intensity actions (PSpeed and PAcc) as evidenced in our study.

Differences in RD, PSpeed, and PAcc could be explained by specificity in playing positions. It was observed in previous research with senior players that guards and forwards had a prevalence in offensive tasks, with emphasis on assists and 3-point field-goals (Sampaio et al., 2006). These indicate that they need to search for free areas outside the three point line, moving around the court from side to side, which explains the distance and speed of their actions (Scanlan et al., 2015a; Reina et al., 2018). However, centers received more impacts, collisions and contacts with opponents, specifically at maximal and supramaximal intensity (Staunton et al., 2018), because their role on the court is related to specific tasks near the basket and into the paint (blocks and defensive/offensive rebounds) (Delextrat et al., 2015). Specific play analysis is needed in order to differentiate the cause of the load and its magnitude, discriminating the high intensity movement actions (guards and forwards) from collisions or contacts (centers).

### Match Periods

According to the present results, time-motion demands relative to match periods showed a significant decrease (*p* < 0.01) in the last quarter. Moreover, intensity variables studied during the first quarter were higher than in all other quarters, with the highest effect size found between the first and second quarter in RD and high-intensity running (relative distance: *p* < 0.05, *d* = −0.78 *moderate effect*; %HIR: *p* < 0.05, *d* = −0.31 *small effect*). This great decrease in RD, HIR and PL could be due to physiological fatigue linked to a peak lactate concentration at half time (Abdelkrim et al., 2007), and an increase in match stoppages (fouls, time outs, etc.) that influence the playing rhythms and overestimate the fatigue-induced performance declines (Linke et al., 2018).

Few studies have reported activity data relative to playing period, and the majority was relative to total distance covered (Oba and Okuda, 2008; Abdelkrim et al., 2010; Scanlan et al., 2012) and total activity frequency (total number of actions performed in all activity types related to time) (Caprino et al., 2012; Scanlan et al., 2012). Abdelkrim et al. (2007) showed a decrease in the amount of high-intensity activity in the last quarter in elite under-19-year-old basketball players (*p* < 0.01; 22.41%) which was lower than the present results in HIR (*p* < 0.01; 28.57%). Other studies confirm these findings, but no data comparability was reported (Abdelkrim et al., 2010; Scanlan et al., 2012). Contrasting results were reported by Delextrat et al. (2015) who failed to find differences between quarters, reporting only a small effect size between the first to the third to last quarter (*d* = 0.1). These contrasting results were found in other previous studies, that seem to be all on female players (Matthew and Delextrat, 2009; Scanlan et al., 2012). Our study showed a decrease in all time-motion variables recorded with significant differences in RD, HIR and PL, from the first to the last quarter that could be associated to players’ fatigue due to the high competitiveness, but it could also reflect their pacing strategies and strategic decisions by coaches (increased time-outs and free-throws) (García-Rubio et al., 2015).

In this respect, it is interesting that the champion team (CFBBP) presented the higher performance decrement between the first and the last quarter in HIR (40.55%), due to the adoption of an all-out strategy that produced a large points difference against the rival (average point difference per quarter in all matches: Q1 = 13.3 ± 2.5; Q2 = 17 ± 4.4; Q3 = 17.6 ± 2.1; Q4 = 15.3 ± 3.51). Thus, this performance decrease could not only be due to greater efficiency, a better technical-tactical level and physiological fatigue, but also to an attempt to achieve a greater points advantage that allows playing with less intensity (Miñano-Espin et al., 2017; Mancha-Triguero et al., 2018), being accentuated in unbalanced matches (Castellano et al., 2011).

### Consecutive Matches Throughout the Tournament

There was a tendency to increase the intensity of the match throughout the tournament. The main finding showed that the intensity increased in the last match of the classification phase (*p* < 0.01). The increase in the intensity of the matches throughout the tournament could be explained due to the eliminatory characteristics of the competition, where the latter games determine the qualification through to finals. To our knowledge, no previous studies have analyzed the match’s external load demands during a tournament in basketball, but this aspect has been studied in other team sports such as hockey and soccer.

Jennings et al. (2012) recorded the Australian elite-male hockey national-team that played six matches in 9 days during the Champions Trophy. In soccer, Odetoyinbo et al. (2007) analyzed three matches during a 5-day winter-period in four elite-level Premier League teams and Arruda et al. (2015) assessed an under-15 years soccer team that played five matches in a 3-day championship. Odetoyinbo et al. (2007) and Jennings et al. (2012) did not find significant differences in time-motion variables of performance throughout these congested-fixture periods. Instead, Arruda et al. (2015) found differences in accelerations per minute, body-load impacts, and body load impacts per minute, but did not find differences in total distance, total distance per minute, number of high-intensity runs, distance covered in HIR and peak running speed. Accelerations per minute decreased during the competition while body-load impacts were higher in the final than in all other matches.

Similarly, the most recent results presented by Arruda et al. (2015) in a same-age population are similar, finding higher body-load impacts in the final match of the championship, but are different in that a decrease in accelerations was observed. In the basketball players analyzed, no differences were found in this variable throughout the tournament, a fact that could be due to the unlimited substitutions rule.

### Limitations

While the results of this study have provided information about the load demands of high-level players across multiple teams, thanks to the use of an advanced tracking system, and considering multiple contextual factors such as team quality, playing position, match periods and the effect of consecutive matches, some limitations to the study must be acknowledged. Because of limited access to individual player information and testing before the tournament, some alternative data analyses could not be performed (e.g., individualized speed and heart rate thresholds). One of the limitations in this research concerns the sample studied. Due to tactical basketball formations, the total sample was distributed unequally by playing positions. Despite this fact, the authors did not influence the natural dynamics of the competition. Finally, data collection was performed under the same conditions throughout the matches (in indoor stadiums and at same time of the day) but the temperature was not controlled.

## Conclusion

The first results on load demands obtained by UWB technology during an elite U’18 basketball tournament indicate that players covered 72.9 ± 2.74 m/min, where 3.44% of actions were at high-intensity running (>16 km/h) and experienced a player load of 1.35 ± 0.09 a.u./min. The best teams played with higher intensity while the worst teams performed a greater volume of movement due to not having the initiative in the match and being less efficient. In the specific players analyzed, playing positions revealed similar demands in accelerometer load and high intensity running. Nonetheless, each role has specific playing demands, where guards and forwards performed more movement while the centers experienced higher impacts. Across the quarters, significant declines were evident following the first quarter, with the greatest decline between the first and second quarters. Finally, load demands increased throughout the tournament, reaching the highest values in the last match of the classification phase that is decisive for success in the competition.

### Practical Applications

The comprehension of the influence of contextual variables analyzed (individual positioning differences, decrease in physical demands throughout quarters, quality of the team and team’s physical behavior throughout the tournament) should be addressed by technical staff for designing conditioning training programs, tactical tasks, match strategy, and recovery protocols during congested fixture periods. Specifically, some practical applications could be considered: (1) Technical staff should study the opponent in order to design the physical load demands in training sessions according to its quality level (a higher-level opponent: more volume of demands; a lower-level opponent: more high intensity actions); (2) Guards and forwards should cover higher distances and reach greater PSpeed and PAcc than centers during training in order to simulate more accurately the match physical requirements; (3) To avoid the effect of fatigue, inter-period recovery strategies should be arranged by medical staff and total playing time should be distributed among the players throughout the match periods; and (4) Technical staff should prescribe correct physical, tactical, and technical demands during the pre-competition period in order to achieve the best performance from the first match of the tournament, since in this research the best performance was shown in the last match of the tournament round.

New devices with microsensor technology are now available for technical staff to quantify the competition and training load demands of athletes. They are non-invasive, reliable, accurate, and portable tools that work in indoor and outdoor conditions. This information is useful for administering individualized training loads, reporting daily feedback data for decision making, and thus achieving optimal performance and maintaining it throughout the season.

## Ethics Statement

The teams’ staffs and tournament managers gave their consent for participation in this research. As all players were over 16 years old, they signed a written consent before the tournament started to give their assent for participation without needing their parents’ permission, and approval was given by the Bioethics Commission of the University (Reg. Code 67/2017). The study was conducted according to the Declaration of Helsinki (World Medical Association, 2013) guidelines.

## Author Contributions

JP-O, FN, and SI conception and design of the study, supervision and editing. CG-C, AB-C, and AH-B data collection. DR-V, CG-C, and AB-C software and database organization. DR-V and JG-R formal analysis. DR-V, CG-C, AB-C, and AH-B writing original draft. JP-O, JG-R, FN, and SI writing review. JG-R and SI funding acquisition. All authors approved the submitted version.

## Conflict of Interest Statement

The authors declare that the research was conducted in the absence of any commercial or financial relationships that could be construed as a potential conflict of interest.

## Footnotes

1 http://www.euroleague.net/final-four/istanbul-2017/news/i/7tcu3xlq5npomb9i/euroleague-basketball-u18-tournament-games-to-be-monitored-using-wimu-pro

2 http://www.adidasngt.com/u18/competition/players
